# Energy conservation characterizes sleep in sharks

**DOI:** 10.1098/rsbl.2021.0259

**Published:** 2022-03-09

**Authors:** Michael L. Kelly, Selwyn P. Collins, John A. Lesku, Jan M. Hemmi, Shaun P. Collin, Craig A. Radford

**Affiliations:** ^1^ School of Life Sciences, La Trobe University, Melbourne, Australia; ^2^ Institute of Marine Science, Leigh Marine Laboratory, The University of Auckland, Auckland, New Zealand; ^3^ School of Biological Sciences, The University of Western Australia, Perth, Australia; ^4^ Oceans Institute, The University of Western Australia, Perth, Australia; ^5^ Oceans Graduate School, The University of Western Australia, Perth, Australia

**Keywords:** elasmobranchs, eye state, metabolism, oxygen consumption, posture, respirometry

## Abstract

Sharks represent the earliest group of jawed vertebrates and as such, they may provide original insight for understanding the evolution of sleep in more derived animals. Unfortunately, beyond a single behavioural investigation, very little is known about sleep in these ancient predators. As such, recordings of physiological indicators of sleep in sharks have never been reported. Reduced energy expenditure arising from sustained restfulness and lowered metabolic rate during sleep have given rise to the hypothesis that sleep plays an important role for energy conservation. To determine whether this idea applies also to sharks, we compared metabolic rates of draughtsboard sharks (*Cephaloscyllium isabellum*) during periods ostensibly thought to be sleep, along with restful and actively swimming sharks across a 24 h period. We also investigated behaviours that often characterize sleep in other animals, including eye closure and postural recumbency, to establish relationships between physiology and behaviour. Overall, lower metabolic rate and a flat body posture reflect sleep in draughtsboard sharks, whereas eye closure is a poorer indication of sleep. Our results support the idea for the conservation of energy as a function of sleep in these basal vertebrates.

## 1. Introduction

Sleep is a ubiquitous behaviour found across the animal kingdom, which is typically characterized by sustained immobility and reduced responsiveness [1]. Despite the vulnerability inherent with sleeping, its persistence across evolutionary time suggests it serves one or more core functions [2]. One hypothesis for such a core function is that sleep serves to conserve energy through enforcing restfulness and lowering metabolic rate relative to wakefulness [1,3,4]. Energy savings during sleep have been reported in diverse animals, including humans [5,6], cats [7], rats [8], birds [9] and fruit flies [10]. It is unknown, however, whether reduced energy expenditure also occurs in sleeping fishes.

Extant sharks represent the earliest group of jawed vertebrates and, therefore, may provide original insight into the evolution of sleep in vertebrates [11]. This rationale is particularly salient following the recent discovery of two sleep states in teleosts [12] and in at least two species of lizard [13,14] that in some respects resemble mammalian and avian non-rapid eye movement (non-REM) and REM sleep [15]. The existence of two sleep states in birds and mammals suggests that each state performs a different, but perhaps complementary, function. Any homology between the multiple sleep states observed in ectothermic vertebrates to that of endothermic vertebrates is unclear.

Recent studies have found that Port Jackson (*Heterodontus portusjacksoni*) and draughtsboard (*Cephaloscyllium isabellum*) sharks are nocturnal with a reduced responsiveness to stimulation while asleep [16,17]. However, as sleep is both a behavioural and physiological state involving multiple components, including changes in eye state, muscle tone, brain activity and metabolism [18], it is necessary to investigate as many sleep components as possible to fully characterize the sleep state, or states, in sharks [19].

Here, in draughtsboard sharks, we assessed changes in metabolic rate (mass-specific oxygen uptake rate or ṀO_2_), via intermittent-flow respirometry, and behaviours associated with sleep in other animals: eye state (open/closed), and body posture (upright/flat) over a 24 h period to determine whether sleep plays a role in energy conservation in sharks.

## 2. Material and methods

### (a) Experimental animals and housing

Seven draughtsboard sharks (766–2705 g in weight) were collected from Hauraki Gulf, north-eastern New Zealand, and were housed in outdoor aquaria under natural light conditions. Animals were fed a diet of pilchards and held for a minimum of two weeks before the commencement of experiments. Food was withheld for at least 48 h prior to the start of experiments to ensure animals reached a post-absorptive state [20,21].

### (b) Intermittent-flow respirometry system set-up

For detailed respirometry methods, see electronic supplementary material [22]. In short, the system comprised an acrylic respirometry chamber submerged within a reservoir tank of flow-through seawater held at constant temperature (17.5°C, 1 µm filtered, UV sterilized). Water was homogenized in the chamber by a pump drawing water from one end and expelling into the other, through a PVC tube [23]. A laptop computer, connected to a Firesting oxygen (O_2_) meter with a contactless sensor spot (Pyroscience, Aachen, Germany) logged oxygen levels. ṀO_2_ measurement cycles were interspersed with flush cycles to ensure a high quality of water (per cent O_2_ range 84–98%).

### (c) Video recording set-up

Continuous, infrared illumination and overhead video recordings of animal behaviour during the 24 h measurement period were achieved following the methods detailed in Kelly *et al*. [16]. For further details on video recording set-up, see electronic supplementary material [22].

### (d) Experimental protocol

Animals were individually placed into the sealed respirometry chamber. Automated, intermittent-flow respirometry and video recordings began a minimum of 48 h later to allow each animal to acclimate to their new conditions before data collection began. Each protocol then lasted 24 h under a 12 : 12 light : dark photoperiod regime.

### (e) Data analysis

**Figure 1.**
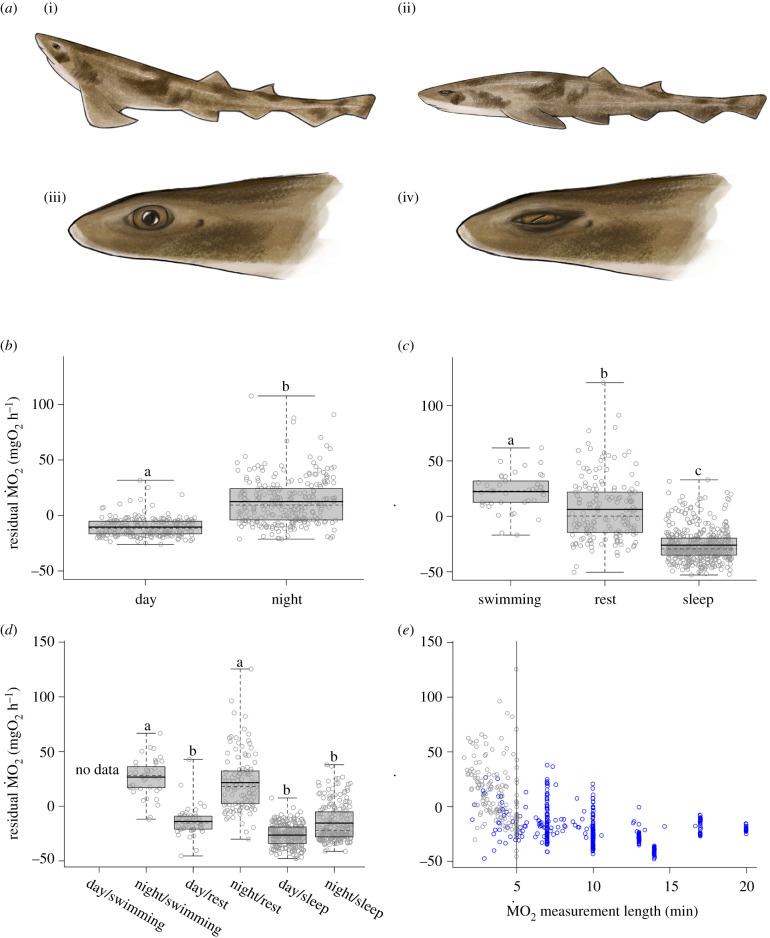
(*a*) Drawings of upright (i) and flat (ii) body postures, and open (iii) and closed (iv) eyes used to score behavioural data from video recordings. (*b*) Box plot of day and night residual ṀO_2_ values (i.e. controlling for body mass) from intermittent-flow respirometry measure periods (all activity states included) over a 24 h period (L : D 12 : 12). (*c*) Box plot of residual ṀO_2_ values across three activity states (irrespective of photoperiod) using subsampled data points from all measure periods with an applied criteria of an *R*^2^ > 0.8 and a length of greater than 90 s. (*d*) Box plot of the residual ṀO_2_ values in (*c*), but partitioned by photoperiod (day, night). (*e*) Regression of subsampled residual ṀO_2_ values against subsample duration (blue indicates sleep; grey denotes rest); all data fit the criteria of an *R*^2^ > 0.8 and a length of greater than 90 s; vertical line indicates 5 min of inactivity. For (*b*,*c*,*d*), solid black lines indicate means; dotted lines denote medians; edges of boxes represent quartiles; whiskers reflect maximum and minimum values; grey circles represent individual samples (random *x*-axis dispersal); significant pairwise contrasts are denoted by the letters a, b and c.

Custom-written software calculated the gradient of the per cent O_2_ decline and the associated residual sum of squares (*R*^2^). ṀO_2_ (mgO_2_ h^−1^) was then calculated from the decline in oxygen saturation. Metabolic rate and behaviour (eye states, posture and activity) data were manually scored second-by-second using the video recordings. Eye states were scored as open or closed and body postures of inactive sharks were scored as flat (lying flat on the bottom of the tank) or upright (sitting perched up on pectoral fins) (figure 1*a*). Activity states were scored as swimming, rest (inactive less than 5 min) or sleep (inactive more than 5 min); the latter has been shown to be associated with reduced responsiveness, and, therefore, a demonstrated reflection of sleep [17]. For details on statistical analyses used, see electronic supplementary material [22].

## 3. Results and discussion

### (a) Sleeping sharks have a lower metabolic rate

Our previous studies showed that draughtsboard sharks are nocturnal [16,17]. Therefore, and unsurprisingly, swimming behaviour and mean ṀO_2_ levels of draughtsboard sharks with an R^2^ > 0.95 (all activity states included) were significantly higher during the night (*t*_12_ = 4.13, *p* < 0.01) (figure 1*b*). However, from these data alone, it remained unclear whether restful sharks were sleeping sharks. To address this question, we sampled the ṀO_2_ data based on activity state (using the criteria of *R*^2^ > 0.8 and bout length of greater than 90 s) to account for varying bout lengths found within each activity state. Shark mean ṀO_2_ levels were significantly lower during sleep (i.e. inactive for at least 5 min) and the highest during swimming (figure 1*c*).

These data were then parsed further to include the expression of each activity state during day and night to reveal whether sleeping animals consistently showed a lower metabolic rate while asleep. During the day, sharks never swam for more than 90 s (with an *R*^2^ > 0.8), therefore, no daytime ṀO_2_ data were available for this state. The level of ṀO_2_ varied between activity states (swimming, rest and sleep) and between day and night (table 1), with ṀO_2_ level recordings again highest during night swimming (figure 1*d*). Metabolic rates were low and similar irrespective of whether sharks slept during the day or night. Variability in ṀO_2_ during night rest was similar to the variability observed during night swimming, suggesting that at least some night rest reflects quiet wakefulness. Accordingly, night rest ṀO_2_ was significantly higher than day rest (Tukey's *post hoc* test: *t*_21_ = −5.06, *p* < 0.01) and during sleep (day: *t*_20_ = −7.18, *p* < 0.01; night: *t*_20_ = 4.97, *p* < 0.01). Conversely, day rest ṀO_2_ was similar to ṀO_2_ during both day sleep (*t*_21_ = 1.39, *p* = 0.73) and night sleep (*t*_22_ = −0.36, *p* = 0.99), which might indicate that sharks fell asleep quicker during the day than during the night (to the effect that at least some day rest should actually be considered to be day sleep). Cube-root transformed residual ṀO_2_ values did not vary with changes in measured rest or sleep duration (figure 1*e*). However, for a given measurement period, the cube-root of residual ṀO_2_ during sleep was between 0.19 and 1.07 less than that observed during rest. These data, therefore, reinforce the results found by Kelly *et al*. [17] that sharks restful for at least 5 min were asleep. Thus, not only do sleeping sharks have reduced responsiveness to stimulation, they also have a lower metabolic rate.

**Table 1.** Mixed effects model showing the effects of activity (swimming, rest and sleep) and photoperiod (12 h day and night) on residual ṀO_2_ values as calculated from a regression of ṀO_2_ and body mass underlying figure 1*d*. In each model, individual was set as a random effect; activity and photoperiod were treated as fixed effects.

effect	nominator d.f.; denominator d.f.	*F*-ratio	*p*-value
ṀO_2_			
activity	2; 21	15.38	<0.01
photoperiod	1; 21	25.08	<0.01
activity * photoperiod	1; 20	5.84	0.03

### (b) Recumbent posture is a better indicator of sleep than eye closure

**Figure 2.**
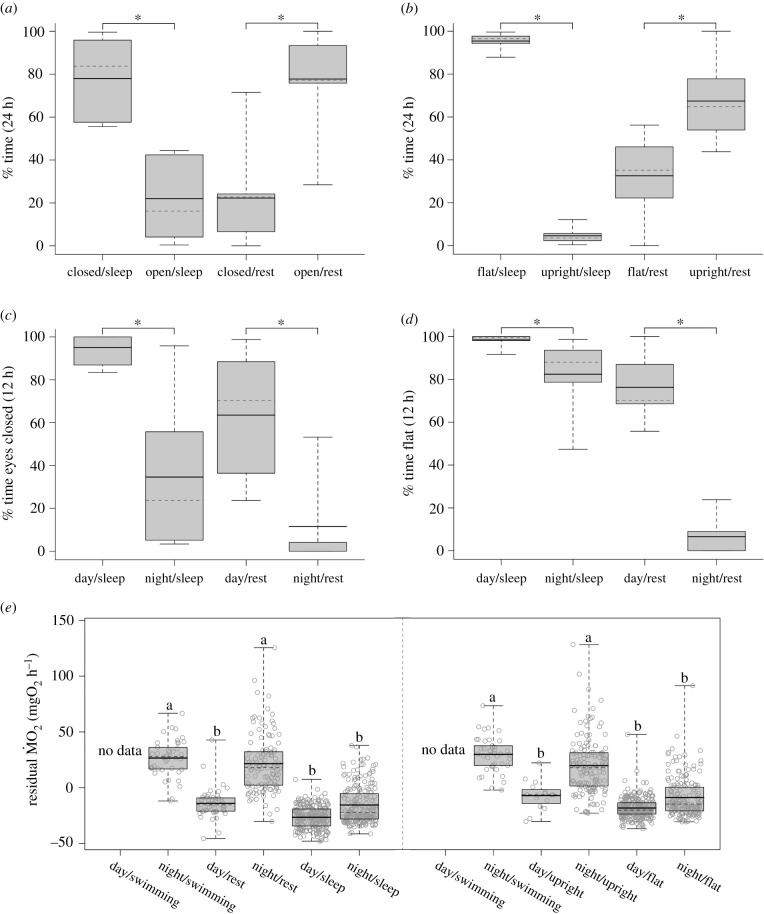
(*a*) Box plot of the per cent time animals were observed with open and closed eyes, and (*b*) in flat and upright postures between sleeping and rest states. (*c*) Box plot showing the per cent of time animals were observed with closed eyes and (*d*) a flat body posture during sleeping and resting states throughout the (12 h) day and night. (*e*) Comparative boxplots of residual ṀO_2_ values across (left) activity states and (right) posture, partitioned by photoperiod, using subsampled data points from all measure periods. For all panels, solid black lines indicate means; dotted lines denote median; edges of boxes represent quartiles; whiskers reflect maximum and minimum values. For (*a*–*d*), significant pairwise contrasts are denoted by asterisks. For (*e*), grey circles represent individual samples (random *x*-axis dispersal); letters a and b denote pairwise contrasts.

While swimming, sharks always had their eyes open. When resting, the eyes were also more likely to be open (z_6,13_ = 161.40, *p* < 0.01) (figure 2*a*). Conversely, during sleep, the eyes were most often closed (z_6,13_ = 353.30, *p* < 0.01). Postural changes were also associated with sleep as sleeping animals adopted a flat body posture (z_6,13_ = 456.60, *p* < 0.01) (figure 2*b*) whereas resting animals sat upright (z_6,13_ = 158.50, *p* < 0.01). This might, at first, suggest that closed eyes and a flat posture reflect sleep, and both are behaviours commonly associated with mammalian sleep [24]. However, upon separating states by photoperiod, we found that eye closure was more common during day sleep (z_6,12_ = 241.74, *p* < 0.01) and day rest (z_6,11_ = 121.09, *p* < 0.01) (figure 2*c*), a behavioural pattern that has also been observed in the large-spotted dogfish (*Scyliorhinus stellaris*) [25]. However, animals that were inactive for more than 5 min (i.e. asleep) during the night had eyes open in approximately 38% of all cases. Taken together, this suggests that eye closure is more likely associated with an external factor, such as the presence of light rather than sleep. Similarly, the proportion of flat body posture was significantly higher during rest (z_6,11_ = 122.49, *p* < 0.01) and sleep states (z_6,12_ = 83.33, *p* < 0.01) throughout the day (figure 2*d*). This supports our ṀO_2_ data that animals inactive for at least 5 min are sleeping. The fact that animals engaged in rest (inactive less than 5 min) during the day spent more time flat also supports the idea that some daytime rest might represent sleep. This might suggest that animals fell asleep faster during the day. It is important to note that night and day ṀO_2_ data partitioned by posture showed a similar pattern to the data partitioned by activity (figure 2*e*). This suggests that both the amount of time spent inactive and body posture are good predictors for sleep in this species.

## 4. Conclusion

The collection of metabolic data via intermittent-flow respirometry in marine fishes, including sharks, is well explored [23,26–31]. Until now, however, no work had directly investigated the metabolic rates of sleeping fishes *per se*. This study highlights that, like in many vertebrates [1], sleep in sharks is associated with reduced metabolic rate. Thus, the hypothesis that sleep is important for energy conservation [3,4] is supported by this study in a primitive vertebrate. By doing so, we have provided the first physiological evidence of sleep in sharks and find support for our published (behavioural) report on sleep in draughtsboard sharks [17]. Sleep is largely unstudied in this diverse group of cartilaginous fishes and future research should focus on other physiological indicators of sleep, such as changes in brain activity, for a more complete portrait of sleep in these vertebrates.

## Data Availability

Datasets and electronic supplementary material are available at datadryad.org. https://doi.org/10.5061/dryad.m37pvmd2z. The data are provided in the electronic supplementary material [22].
